# Underlying the Mechanisms of Doxorubicin-Induced Acute Cardiotoxicity: Oxidative Stress and Cell Death

**DOI:** 10.7150/ijbs.65258

**Published:** 2022-01-01

**Authors:** Chun-Yan Kong, Zhen Guo, Peng Song, Xin Zhang, Yu-Pei Yuan, Teng Teng, Ling Yan, Qi-Zhu Tang

**Affiliations:** 1 Department of Cardiology, Renmin Hospital of Wuhan University, Wuhan 430060, RP China; 2 Hubei Key Laboratory of Metabolic and Chronic Diseases, Wuhan 430060, RP China

**Keywords:** Doxorubicin, cardiotoxicity, oxidative stress, cell death

## Abstract

Cancer is a destructive disease that causes high levels of morbidity and mortality. Doxorubicin (DOX) is a highly efficient antineoplastic chemotherapeutic drug, but its use places survivors at risk for cardiotoxicity. Many studies have demonstrated that multiple factors are involved in DOX-induced acute cardiotoxicity. Among them, oxidative stress and cell death predominate. In this review, we provide a comprehensive overview of the mechanisms underlying the source and effect of free radicals and dependent cell death pathways induced by DOX. Hence, we attempt to explain the cellular mechanisms of oxidative stress and cell death that elicit acute cardiotoxicity and provide new insights for researchers to discover potential therapeutic strategies to prevent or reverse doxorubicin-induced cardiotoxicity.

## Introduction

Cancer is a systemic disease accompanied with severe damage to multiple organs and distant invasion, resulting in high morbidity and mortality and adding marked threats and burdens to the public 1. DOX is efficacious as a broad-spectrum chemotherapeutic drug and has attracted much attention. However, DOX is viewed as a double-edged sword due to its severe dose-dependent cardiotoxicity side effects. The toxicity of DOX causes myocyte damage and ultimately leads to the pathological cardiomyopathy 2. Evidence shows that the incidence of DOX-induced cardiotoxicity is dependent on several factors, such as the cumulative dose of DOX, dosing regimen and age^3^. DOX-induced cardiotoxicity can be acute, subacute or chronic. DOX-induced acute cardiotoxicity usually occurs 2-3 days after DOX administration, and the incidence rate is approximately 11% 4. Typical histological alterations of DOX-induced acute cardiac injury include cytoplasmic vacuolation and sparsity and interruption of myofibrils 5, 6. Current studies are giving increased attention to DOX-induced acute cardiotoxicity. Therefore, the subsequent part of the review focuses on acute cardiotoxicity induced by DOX.

DOX causes the increased absorption of oxygen and produces multiple types of reactive oxygen species (ROS), leading to oxidative stress 7. Data show that DOX-induced ROS generation can trigger the pathological sarcoplasmic reticulum (SR)/Ca^2+^ leakage, DNA damage and interruption of autophagic flux, causing ultimately lipid peroxidation-dependent ferroptosis and many other types of regulated cell death 8-11. Increasing evidence has provided insight into the importance of regulated cell death pathways in DOX-induced cardiotoxicity^12^, ^13^. These aspects of DOX-induced cardiotoxicity have been well summarized previously 14, 15. However, the precise mechanism network between DOX-induced oxidative stress and regulated cell death remains unclear and the clinical approaches are rare 16. In this review, we summarize evidences supporting the leading roles of oxidative stress in subcellular disorder, and ultimately regulated cell death in the development and maintenance of DOX-induced cardiotoxicity and the potential benefits of targeting oxidative stress and regulated cell death to provide a new perspective on preventing DOX-induced cardiotoxicity to readers.

## Oxidative stress in response to DOX

DOX-induced oxidative stress is deemed the major cause of cardiotoxicity, which is identified as an imbalance in the levels ROS and reactive nitrogen species (RNS), and is linked to dysregulation of antioxidants, subsequent destruction of subcellular structure and regulated cell death^17^, ^18^.

DOX is reduced to semiquinone through one-electron reduction, while oxidases, such as nicotinamide adenine dinucleotide phosphate (NADPH) oxidases (NOXs), and uncoupled nitric oxide synthases (NOSs) are electron donors. Semiquinone can autoxidize in the presence of oxygen and then produce superoxide anions 19-21.

### The role of NOXs in DOX-dependent oxidative stress

**Figure 1:**
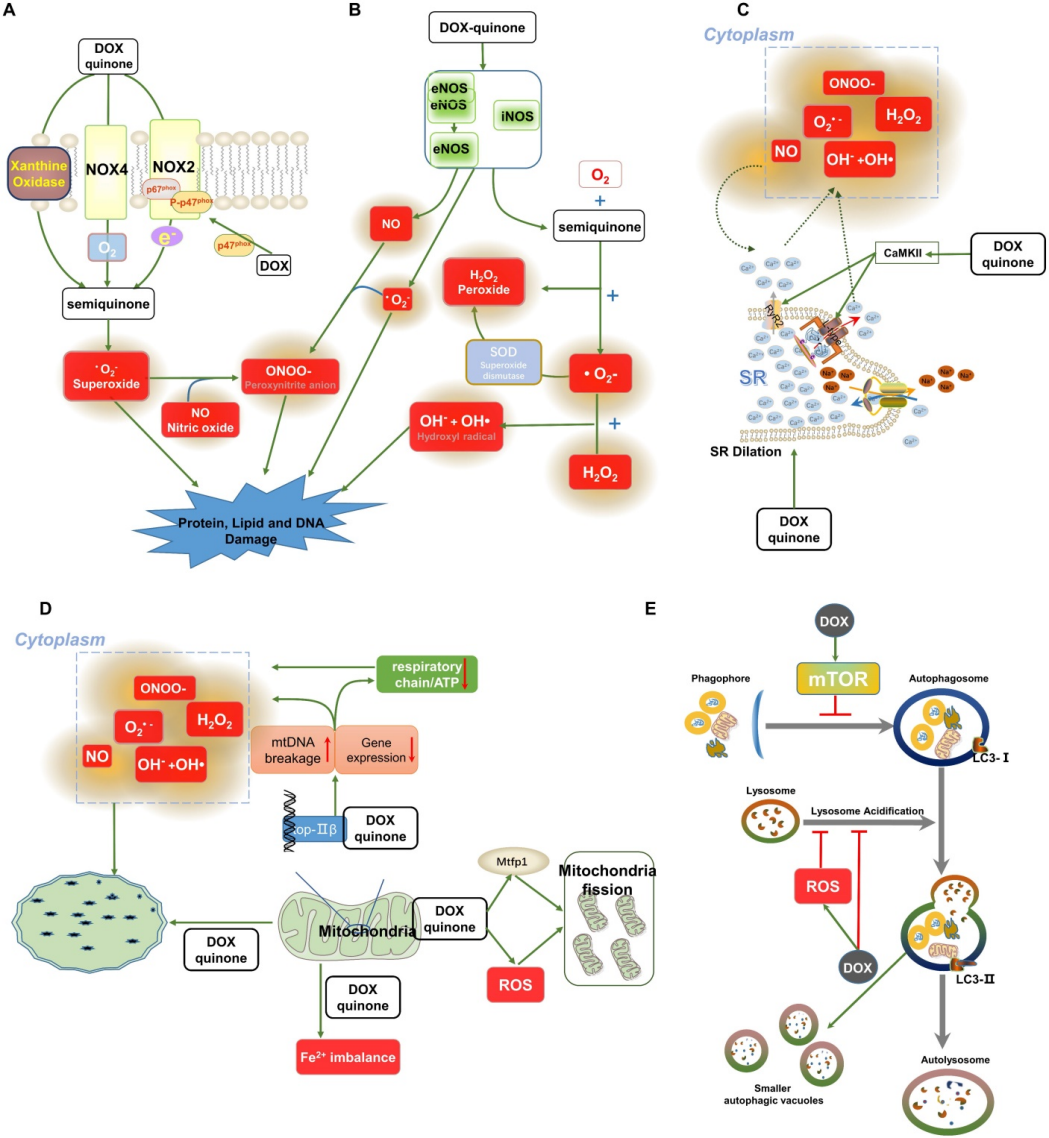
Oxidative stress-related signalling pathways involved in DOX-induced cardiomyopathy. (A-B) Roles of NOXs and NOSs in the molecular transformation of DOX. DOX is reduced to a semiquinone by NOSs and NOXs along with the generation of ROS, and RNS includes •O_2_**^-^**, H_2_O_2_, NO, OH^-^ and OH•. NO can react with •O_2_**^-^** or H_2_O_2_ to produce ONOO-. In addition, xanthine oxidase can also cause the generation of ONOO- via the stimulation of DOX. (C) Data show that loss of intracellular Ca^2+^ homeostasis induced by DOX contributes to the generation of free radicals. DOX phosphorylates CaMKII and prolongs the opening time of Ca^2+^ channels, leading to Ca^2+^ leakage. The increased concentration of Ca^2+^ induces the production of ROS. (D) DOX influences the integrity of mitochondrial DNA by combining with TOP2β, damaging mitochondrial function and ultimately leading to the excessive accumulation of ROS. (E) A brief overview of DOX-induced inhibition of autophagy. Abbreviations: NOXs, NADPH oxidases; NOSs, Nitric oxide synthases; ROS and RNS, Reactive oxygen and nitrogen species; •O_2_**^-^**, Superoxide; H_2_O_2_, Peroxide; SOD, Superoxide dismutase; NO, Nitric oxide; OH^-^ and OH•, Hydroxyl radical; ONOO-, Peroxynitrite anion; SR, Sarcoplasm reticulum; CaMKII, Ca/calmodulin-dependent protein kinase II; TOP2β, Topoisomerases-2β. The activation signal is indicated by the green stripe arrows, and the inhibition signal is indicated by the red stop symbols.

Several studies have demonstrated that DOX induces changes in NOXs, which transfer electrogenic H^+^ currents across phospholipid bilayers and convert oxygen into superoxide, leading to ROS production 22, 23. The NOX family comprises seven members (NOX1-5 and DUOX1-2). In mammalian hearts, NOX2 and NOX4 are predominant and contribute to the accumulation of ROS in response to DOX exposure (Figure 1A) 24. Generally, NOX2 is quiescently located in the cell membrane, working as enzymes and transferring electrons from the cytosol into various intracellular and extracellular compartments. Once activated by DOX stimulation, NOX2 participates in the reduction of DOX and generation of superoxide anion (•O_2_**^-^**) and hydrogen peroxide (H_2_O_2_)^25^. Consistent with this, our previous research demonstrated that DOX promotes NOX2 expression and leads to phosphorylation of p47phox, a cytoplasmic subunit of NOX2. The membrane translocation of phosphorylated p47phox activates NOX2 and promotes the production of ROS induced by DOX 26. In addition, we confirmed that the administration of irisin, an antioxidant, inhibits the expression of p67phox of NOX2 and suppresses the activity of NOX2, therefore protecting against DOX-induced oxidative stress in the heart 27. It was further confirmed that the absence of NOX2 attenuates cardiac •O_2_**^-^** production and protects against DOX-induced cardiac injury 26, 28. However, there are contradictory reports regarding the role of NOX2 in cardiovascular diseases. NOX2-deficient mice develop severe glucose metabolism disorders in response to 16 weeks of a high-fat diet, while overexpression of NOX2 causes transient enhancement of acute contractility under angiotensin Ⅱ stimulation through NOX2-mediated Ca^2+^ uptake 29, 30. These conflicting results may be explained by the different roles of NOX2 in different cardiac cell populations under different frequencies and magnitudes of cardiac stimuli. Additionally, DOX also activates the mitochondria-targeting signal of NOX4 and induces NOX4 to interact with mitochondria, generating ROS 31. Overexpression of NOX4 promotes the activation of inflammasomes, resulting in pyroptosis in DOX-treated hearts 32. Conversely, NOX4 inhibition with small interfering RNA (siRNA) attenuated DOX-induced cardiac injury 33. The data from our lab indicated that osteocrin treatment markedly inhibits the generation of ROS by inhibiting the expression of NOX4 rather than NOX2 in DOX-treated cardiomyocytes 34. Although accumulated lines of evidences have indicated the role of NOXs in DOX-induced oxidative stress, no clinically available NOXs inhibitors have been developed. Future experiments will be required to determine whether any NOXs inhibitors hold true in clinical settings.

### NOSs in DOX-induced oxidative stress

Experimental studies exploring protection against DOX-induced cardiotoxicity depending on the use of phenylalanine-butyramide, levosimendan and antioxidants (vitamin C) to reduce oxidative stress and nitrosative stress have reported altered production of NOSs, showing that NOSs are engaged in DOX-induced cardiotoxicity (Figure 1B)^23^, ^35^, ^36^.

These findings had led to investigations showing that DOX stimulates expression of the inducible NOS (iNOS) enzyme to generate nitric oxide (NO) and increase the production of H_2_O_2_ and •O_2_**^-^**, while the interaction between NO and •O_2_**^-^**- generates free radical-peroxynitrite anion (ONOO-)^35^, ^37^, ^38^. Accumulation of these highly reactive radicals contributes to oxidative stress induced by DOX and causes DNA damage 39. Evidence indicates increased iNOS protein and mRNA expression in the myocardium in response to DOX treatment. Increased generation of NO and •O_2_**^-^** was detected after DOX treatment, triggering oxidative stress in the heart. To explore the role of iNOS in DOX-induced ROS production, iNOS knockout mice were used. Data showed that the absence of iNOS attenuates DOX-induced generation of NO and •O_2_**^-^** and subsequent cell damage 40. Indeed, conflicting results regarding DOX-iNOS were observed in studies. Most studies showed that iNOS-deficient mice inhibites DOX-induced oxidative stress and nitrosative stress in the myocardium, whereas Cole et al reported that iNOS-deficient mice exhibit reduced production of NO, which causes more extensive cellular damage by DOX. The diametrically opposite results of these two experiments might be due to differences in the genetic background of the mice. Different types of mice might experience different outcomes during DOX-induced cellular damage, given that iNOS in different genetic backgrounds of mice modulates diverse levels of ROS and RNS 40, 41.

eNOS catalyses the biological synthesis of NO and acts as another major mediator of DOX-dependent ROS 20, 42. Evidence had shown that DOX treatment increases the ratio of monomeric/dimeric eNOS, which facilitates the generation of •O_2_**^-^** rather than NO and ROS/RNS in a positive feedback loop. This eNOS-dependent ROS/RNS accumulation is associated with cell damage induced by DOX 36, 43, 44. In another study, eNOS-deficient mice showed a marked reduction in the production of ROS and the frequency of cell death, whereas eNOS-TG mice potentiated DOX-induced cardiotoxicity 45. Consistent with this, Laura Tedesco et al suggested that administration of an α5 mixture antagonized the reduction in eNOS phosphorylation, inhibiting ROS production induced by DOX 46. However, evidence is also contradictory concerning the actual role of eNOS in DOX-induced cardiotoxicity. The investigation suggested that eNOS-deficient female mice experienced aggravated DOX-induced oxidative stress and cellular damage 47. These conflicting results might be explained by the sex and hormone differences in the experiments 48.

### The role of xanthine oxidases and peroxisomes in DOX-dependent oxidative stress

Xanthine oxidases, which play a similar role in DOX-dependent oxidative stress as NADPH oxidase and NOSs, catalyse the reduction of DOX, triggering oxidative stress in myocardial tissue 40. As noted in previous studies, one-electron DOX reduction, a key process in oxidative stress development, is catalysed by xanthine oxidases, and its inhibitor febuxostat has been demonstrated to effectively inhibit the formation of ROS, thereby blocking cardiotoxicity associated with DOX (Figure 1A). However, it has not been used in clinical practice 49, 50.

Peroxisomes are reported to maintain redox homeostasis, for instance, by participating in β‐oxidation of fatty acids and the detoxification of H_2_O_2_. A previous study demonstrated increased number of peroxisomes in DOX-treated neurons. The upregulation of peroxisomes enhanced the generation of ROS and contributed to neurotoxicity 51. However, few studies have specifically assessed the role of peroxisomes in DOX-induced cardiotoxicity. Therefore, the utility of pharmacologically targeting peroxisomes in DOX-induced cardiotoxicity remains to be explored.

### The decrease in antioxidants

The heart expresses lower levels of endogenous antioxidant enzymes, including superoxide dismutase (SOD), glutathione peroxidase (GPx), catalase (CAT) and glutathione (GSH), making it more vulnerable to DOX-induced cardiac injury 52, 53. ROS/RNS produced by DOX treatment decrease the expression of antioxidants and impair their activities in a time- and dose-dependent manner 7, 54. Evidence indicated that a significant reduction in SOD and GPx was detected after 24 hours of a low dose of DOX administration 54. The role of these antioxidants in DOX-related cardiac injury was revealed by the use of transgenic mice. Overexpression of these antioxidants (GPx1, Mn-SOD or CAT) enhanced their abilities to remove ROS and reduced DOX-induced acute cardiac injury 55-58. Conversely, DOX-induced ROS accumulation was increased in GPx-1-deficient mice, and the absence of GPx made the heart more vulnerable to DOX 59. Several drugs that serve as ROS scavengers had been found to enhance the ability of antioxidants and attenuate ROS production but have little effect in clinical practice 60, 61. The major reasons for the ineffectiveness of antioxidants might be their low bioavailability and the low overall scavenging ability of ROS, suggesting a limitation of protection against DOX-induced oxidative stress using ROS scavengers or antioxidant supplementation 57, 58.

### Oxidative stress and Nrf2 in cardiotoxicity

Nuclear factor erythroid 2 like 2 (Nrf2) functions as an oxidative stress sensor to regulate adaptive response to xenobiotic compounds 62. Nrf2 is negatively regulated by Kelch Like ECH associated protein 1 (Keap1)-dependent proteasomal degradation. Degradation of Keap1 leads to constitutive activation of Nrf2, which enters into nucleus and regulates a set of antioxidant enzymes, like glutathione S-transferase (GST), heme oxygenase-1 (HO-1), and NAD(P)H quinone dehydrogenase 1 (NQO1) 63. In DOX-induced cardiotoxicity, Nrf2 was largely downregulated, resulting in the lack of antioxidant enzymes expression 64. Finding from the animal experiments revealed that activation of Nrf2 reduced DOX-induced oxidative impact to cardiomyocytes 65. Consistent with this, Nrf2-deficient mice aggregated DOX-induced oxidative stress in the heart and abolished the protective effect of follistatin-like 1 as a Nrf2 agonist 66. Our previous study also demonstrated that Nrf2 protein expression could be restored by irisin, which was responsible for the protective role of irisin in DOX-induced cardiotoxicity 27.

### Acetylation in DOX-dependent oxidative stress

Acetylation is an evolutionarily conserved post-translational modifications with acyl-CoA. Protein deacetylation in human relies on NAD-dependent silent information regulator factor 2 (Sir2)-related deacetylates (SIRT, including human Sir2 homolog from SIRT1 to SIRT7) 67-69. Evidences found that DOX treatment significantly suppressed SIRT1 deacetylase activity and protein level 9, 70. Our pervious researches confirmed that the activation of SIRT1 could protect against the generation of ROS induced by DOX, however, SIRT1 deficiency abolished the antioxidant and anti-apoptotic capacities of drugs in DOX-treated mice 70, 71. Consistently, overexpression of SIRT1 restored the de-ubiquitination of p53 induced by DOX and reduced the activation of caspase-3 and apoptotic DNA fragmentation 72, 73. Similarly, SIRT2 and SIRT3 were suppressed in DOX-treated cardiac tissue, whereas the overexpression of them rescued the generation of intracellular ROS and myocardial apoptosis through promoting the expression levels of HO-1, NQO1, Keap1 and antioxidant enzymes 64, 74, 75. Notwithstanding the explorations of the compound for activation of SIRT deacetylates, there is still a lack of efficient drugs of SIRT activator for clinical therapy.

## Destruction of subcellular structure induced by DOX

DOX causes cytoplasmic vacuolation and disorder of myofibrils in the heart, destroying the structure of subcellular organelles, including the SR, mitochondria and autolysosomes 6, 10, 76, 77. Electron microscopy revealed severe dilation of SR and abundant sarcoplasmic vacuoles in the hearts of DOX-treated mice 5, 6. DOX-induced morphological abnormalities of the SR also led to ion disturbances (Figure 1C). Studies have shown that DOX increases Ca/calmodulin-dependent protein kinase II (CaMKII) phosphorylation, and Ca^2+^-dependent activation of CaMKII and pathological SR/Ca^2+^ leakage were detected after CaMKII activation in response to DOX exposure. Phosphorylation of CaMKII enhances the opening of cardiac ryanodine receptor 2 (RyR2) clusters and leads to SR/Ca^2+^ leakage 77. In turn, the increased concentration of Ca^2+^ in the cytoplasm causes the generation of ROS in a vicious cycle, which further impairs the structure of the SR 8.

Mitochondrial impairment is another typical pathological alteration of DOX-induced cardiomyocyte injury (Figure 1D). DOX triggers the formation of vacuoles distributed in the perinucleus and cytoplasm, which are highly suspected to be swollen mitochondria 6. Upon DOX stimulation, the mitochondria proliferates abnormally due to mitochondrial DNA (mtDNA) breakage, an imbalance of mitochondrial fission/fusion, interruption of the respiratory chain and loss of mitochondrial iron homeostasis 5, 9. Generally, DOX exerts anticancer properties via topoisomerase (TOP, especially Top1 and Top2) inhibition 78. Unlike tumour cells which preferentially express Top2α, cardiomyocytes selectively express Top2β. Evidence had shown that Top2β binds to DOX and mtDNA, resulting in the formation of DOX-Top2β-mtDNA complexes in the heart. The formation of DOX-Top2β-mtDNA complexes decreases the transcriptome involved in mitochondrial biogenesis and increases the generation of ROS 79. Data indicated that the generation of ROS is significantly reduced by the absence of Top2β, confirming that DOX-induced ROS production depends on Top2β 80. Evidence had shown that DOX treatment increases ROS production, which in turn causes a decrease in mtDNA copy number and DNA double-strand breaks 9. The increased ROS production in response to DOX treatment is strongly linked to the imbalance of mitochondrial fusion and fission, which regulate the population and functions of mitochondria. Evidence had indicated that mitochondrial fission protein 1 (Mtfp1, a protein involved in mitochondrial fission) is upregulated in response to DOX treatment. The higher expression levels of Mtfp1 lead to excessive mitochondrial fission and triggered initiation of apoptosis; however, knockdown of Mtfp1 suppresses the percentage of fission and facilitated the fusion of mitochondria 81. The excessive oxidative stress caused by DOX is also linked to mitochondrial membrane potential. Ana R. Coelho et al demonstrated that DOX has a robust attraction to the mitochondrial membrane, which promotes mitochondrial dysfunction, causing lipid peroxidation and leading to downregulation of ROS-detoxifying mechanisms by stimulating the* de novo* formation of respiratory chain components 82.

DOX also impairs autophagic flux, which serves as an ATP supply through the process of degradation and circulation of intracellular molecules. DOX stimulation was confirmed to inhibit the initiation of autophagy and interfere with the formation of autolysosomes 10. DOX stimulates the phosphorylation of mammalian target of rapamycin (mTOR), which suppresses the formation of autolysosomes and inhibits autophagic flux 83, 84. The inhibition of autophagic flux caused by DOX leads to a decrease in the content of autolysosomes and autophagosomes in the hearts 85. However, this finding had been challenged. Electron microscopy demonstrated the opposite result: DOX treatment led to a significant increase in autolysosome number with no changes in autophagosomes 10. These conflicting results can be explained by the fact that DOX not only affects the initiation of autophagy but also influences the maturation of autolysosomes. The formation of autolysosomes is accompanied by the fusion of lysosomes and autophagosomes^10^, ^85^. DOX-dependent ROS decreases enzymatic activity in lysosomes and impairs lysosomal acidification, interdicting the autolysosome formation process 10, 86. The decreased formation of autolysosomes leads to a slight increase in autophagosomes, ultimately leading to normalized autophagosome numbers in response to DOX stimulation (Figure 1E). The accumulation of autolysosomes might be explained by DOX-induced abnormal lysosomal acidification leading to the formation of smaller autolysosomes.

## Cell fate

Several genetically defined cell death pathways exist, including apoptosis, autophagy, pyroptosis and ferroptosis, which are involved in DOX-induced acute cardiotoxicity. Next, we discuss the current cell death studies in DOX-induced acute cardiotoxicity.

### Apoptosis

**Figure 2:**
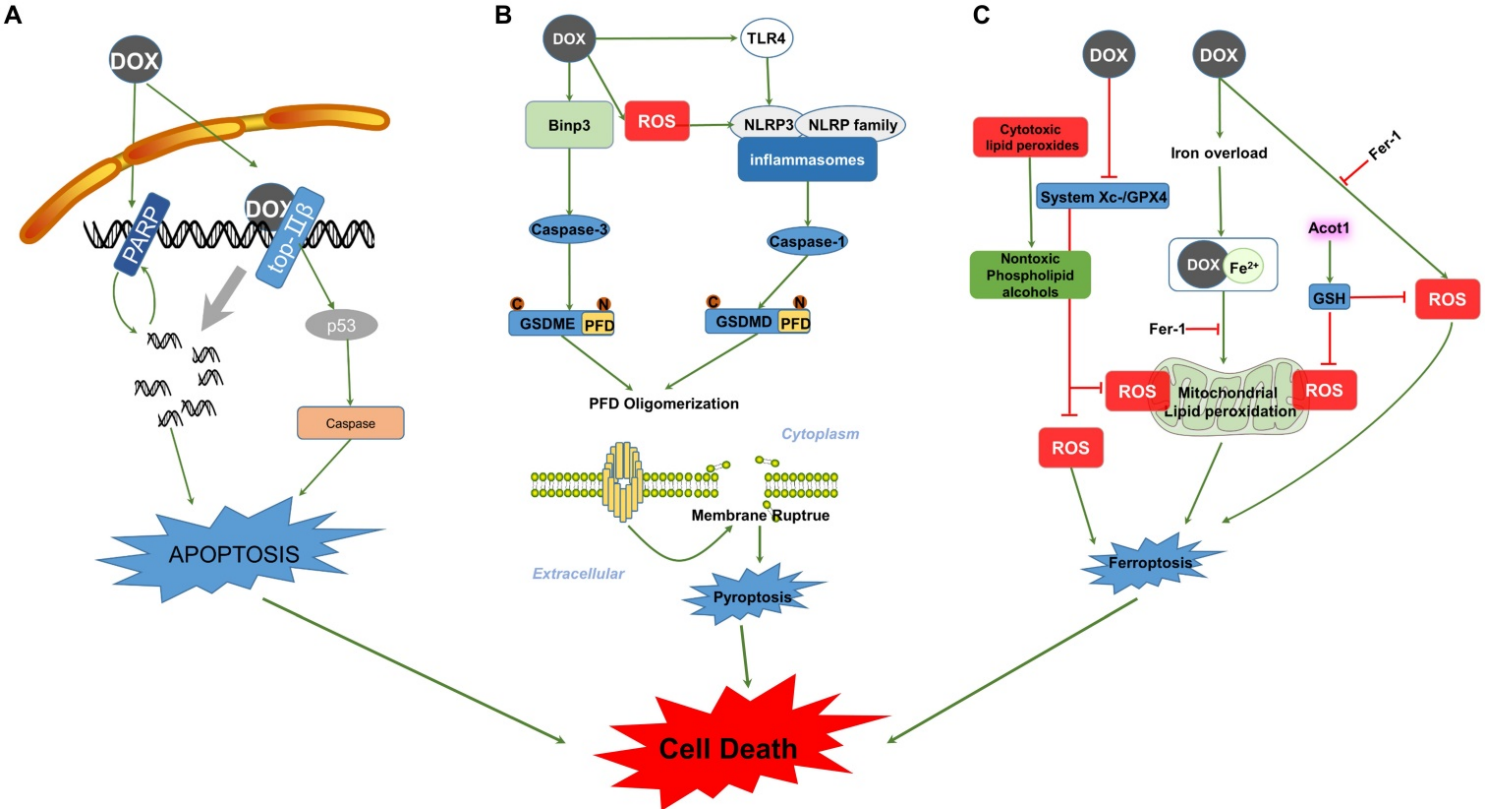
Signalling pathways involved in DOX-induced cell death. (A) The left side of the schematic diagram shows the function of PARP in cell apoptosis, which can be activated by DOX-induced DNA breaks in positive feedback and results in cell apoptosis. DOX also interacts with Top2β, forming Top2β-DOX-DNA complexes and leading to DNA apoptosis in a p53-dependent manner. (B) The picture illustrates the major steps constituting the process of pyroptosis in the caspase-3/GSDME and inflammasome/GSDMD pathways. DOX induces the activation of caspase and cleavage of PFD from the N-terminus of the GSDM family. PFD forms large pores in the membrane through oligomerization, resulting in membrane rupture and pyroptosis. (C) DOX-induced ferroptosis is dependent on lipid peroxidation and Fe^2+^ imbalance. Abbreviations: PARP, poly (ADP-ribose) polymerase; Top2β, Topoisomerases-2β; Binp-3, BH3-only protein Bcl-2/adenovirus E1B 19-kDa-interacting protein 3; TLR4, Toll like receptor 4; NLRP3, NOD-like receptor; GSDMD, Gasdermin D; GSDME, Gasdermin E; PFD, Pore-forming domain; GPX, Glutathione peroxidase; ROS, Reactive oxygen species; Acot1, Acyl-CoA thioesterase 1; Fer-1, Ferrostatin-1; GSH, Glutathione. The activation signal is indicated by the green stripe arrows, and the inhibition signal is indicated by the red stop symbols.

Evidence had shown that a higher level of Top2β renders cardiomyocytes more sensitive to DOX and causes increased DNA damage; in contrast, cardiomyocyte-specific deletion of Top2β suppresses the apoptosis and cardiotoxicity mediated by DOX 80, 87. The formation of DOX-Top2β-DNA complexes is the first step of apoptosis induced by DOX. Subsequently, these complexes cause DNA damage and induce genotoxic stress by promoting the phosphorylation of p53 88. DOX treatment induces p53-dependent caspase activation, leading to cardiomyocyte-apoptosis 89. The p53 inhibition attenuates DOX-induced acute cardiotoxicity in dominant-interfering p53-expressing mice. During the acute stage, preserved cardiac function and decreased levels of apoptosis were detected in response to p53 inhibition; in contrast, mice that express dominant-interfering p53 in cardiomyocytes exhibited higher levels of apoptosis during the late stage of DOX treatment 90. Interestingly, p53 mediated cardiomyocyte apoptosis and simultaneously affected the differentiation of cardiac progenitor cells (CPCs) towards cardiomyocytes induced by DOX treatment. This result indicates that the presence of p53 is necessary for the transition of CPCs under DOX stimulation, while p53-dependent apoptosis is absent in CPCs 91. However, the DOX-induced and p53-dependent differentiation of cardiomyocytes committed to CPCs was relatively small 91, 92. Therefore, the impact of p53 inhibition on DOX-induced apoptosis differed due to the schedule and cessation of drug treatment, and the subpopulation of cells in the heart and these observations indicate that p53 activation plays a central role in DOX-induced apoptosis (Figure 2A).

In addition to Top, poly (ADP-ribose) polymerase (PARP) enzymes also contribute to DOX-induced apoptosis in the heart. DOX-induced oxidative stress activates the release of PARP cleavage and leads to apoptosis and consequently, cell loss. PARP-1 inhibition prevents DOX-induced apoptosis in the myocardium *in vivo* 93. Consistent with this, two other studies also indicated that PARP inhibitors exert protective effects against apoptotic cell damage in response to DOX treatment in mice 94, 95. However, a recent study showed an adverse effect of PARP-1 inhibitor as increased the generation of DNA strand breaks and apoptotic myocyte death 81. This inconsistency may be explained by the nonspecific effects of these PARP-1 inhibitors.

### Pyroptosis

Pyroptosis is another form of cell death that takes part in the progression of DOX-induced cardiotoxicity (Figure 2B). DOX increases expression of BH3-only protein Bcl-2/adenovirus E1B 19-kDa-interacting protein 3 (Bnip3) in myocytes, inducing activation of caspase-3 and cleavage of Gasdermin E (GSDME) in cardiomyocytes and resulting in membrane rupture and pyroptosis 13, 96. Data revealed that either knockdown of GSDME with siRNA or blockade of caspase-3 attenuated approximately 50% of DOX-induced pyroptosis in macrophages 97. In addition, DOX activated Toll-like receptor 4 (TLR4) and promoted the formation of NLRP3 inflammasomes to activate caspase-1 and gasdermin D (GSDMD), mediating the occurrence of pyroptosis 98, 99. DOX also activated NLRP3 in a TLR4-independent but ROS-dependent manner. This result indicates that DOX treatment induces overexpression of NOX1/NOX4 and triggers mitochondrial fission, promoting the accumulation of ROS and subsequent NLRP3 inflammasome activation and pyroptosis in the heart 32. Moreover, NLRP3- and caspase-1-deficient mice were confirmed to attenuate DOX-induced pyroptotic cell death, suggesting a critical role of NLRP3 inflammasomes and caspase-1 in DOX-induced pyroptotic cell death 32. However, additional studies are still needed. Identifying a regulator that suppresses both NLRP3/caspase-1 and caspase-3/GSDME might be a potential therapeutic approach for DOX-induced cardiotoxicity.

### Ferroptosis

Ferroptosis is a newly identified type of cell death involved in DOX-induced cardiotoxicity (Figure 2C). DOX treatment facilitates the release of Fe^2+^ and forms a DOX-Fe^2+^ complex in mitochondria, which led to ROS accumulation and resulted in lipid peroxidation-dependent ferroptosis 11. Consistent with this, scavenging Fe^2+^-initiated lipid peroxidation with ferrostatin-1 (Fer-1) prevented DOX-induced ferroptosis in mitochondria 100, 101. Acyl-CoA thioesterase 1 (Acot1) is an important enzyme in lipid metabolism and is inhibited by DOX treatment. Evidence shows that overexpression of Acot1 elevated antioxidants (GSH) and inhibited lipid peroxidation in cardiomyocytes, rescuing levels of ferroptosis induced by DOX. While Acot1 knockdown made the heart more vulnerable to DOX-induced ferroptosis, this exacerbation caused by Acot1 knockdown was rescued by Fer-1 cotreatment^101^. These observations suggest an important role of Fe^2+^-dependent mitochondrial ROS in ferroptosis. Additionally, studies indicate that DOX inhibits expression of system Xc-/GPx4 and interrupts the conversion of toxic cytotoxic lipid peroxides to nontoxic phospholipid alcohols, subsequently leading to ferroptosis 12, 102, 103. Conversely, GPx4 overexpression suppressed the accumulation of lipid peroxidation and prevented the progression of ferroptosis induced by DOX; in contrast, GPx4 deletion exacerbated DOX-induced ferroptosis 104. This study also indicated that DOX-induced ferroptosis was caused by the formation of the DOX-Fe^2+^ complex but not the DOX-Fe^3+^ complex and that an iron chelator might play an important role in suppressing lipid peroxidation-dependent ferroptosis in DOX-treated hearts. However, the reduction of Fe^3+^ to Fe^2+^ makes it difficult for iron chelators to inhibit Fe^2+^-initiated ferroptosis.

## Multi-omics reflecting a whole picture of mechanisms behind DOX-induced cardiotoxicity

With the advent of techniques, comprehensive omics strategies come into vision and multi-omics analysis has been applied in DOX-associated cardiotoxicity for high-throughput data of every single cell, providing beneficial for diagnosis and therapy 105-107.

RNA-sequencing analysis (RNA-seq) and proteomics analysis were performed in hearts detecting thousands of genes or proteins and distinguished significantly differential expression groups (DEGs) 108-111. The analysis of DEGs with KEGG-pathway enrichment or gene ontology (GO) approaches clustered the dysregulated genes in lipid metabolism, cell growth and death, cell motility, cellular community and other signals about genetic information, metabolism and organismal systems 101, 108, 112. And the proteomic data helped to screen the protein which is sensitive and related to the pathogenesis of cardiac toxicity induced by DOX. Subsequent bioinformatics analysis given the fact the altered peptides were closely associated with mitochondrial function, energy generation, oxidation-reduction process and oxidative phosphorylation 113. Bioinformatics profiling of integrated multi-omics screened common pathways involved in DOX-induced cardiotoxicity, which is indicated to be associated with energy metabolism, amino acid metabolism, fatty acid metabolism, and enzyme activities 106, 107. However, the differences in experimental models, grouping methods, analysing approaches, and screening conditions lead to various database. The computational multi-omics approaches never stop renewing and the exploration of the mechanisms behind DOX-induced cardiac injury will go on.

## Future challenges

The invention of DOX has brought about precious opportunities to cure cancer, but its toxic side effects on the heart can be lethal, restricting its clinical employment. Numerous studies have been performed to investigate the underlying mechanisms of DOX-induced cardiomyopathy, which has been confirmed to be both complex and multifactorial. It has been demonstrated that oxidative stress is the leading cause of the pathophysiology of DOX-induced cardiomyopathy. However, there are still some open issues in this area: (1) Our knowledge regarding the mechanisms of DOX-induced acute cardiac injury predominantly comes from experiments performed in cell culture systems or global knockout mice, which cannot completely mimic the pathological process of DOX-induced cardiotoxicity in human beings. Therefore, whether these findings can translate into clinical practice remains unclear. (2) To date, there are no effective interventions against DOX-induced cardiotoxicity except for dexrazoxane. Strategies targeted ROS or cell death cannot effectively work in their clinical practice. This is due to low scavenging efficacy of ROS and secondary reactions with other biomolecules. From this aspect, whether attenuation of oxidative stress and apoptosis will become a promising therapeutic approach is unknown. (3) Additionally, few researches are launched around cardiac fibroblast in DOX-treated mice, remaining the role of cardiac fibroblast and cardiac fibroblast-derived factors in DOX-induced cardiotoxicity unclear. Another decade may have to elapse before a better understanding of the mechanism of DOX-induced cardiotoxicity is achieved.

## References

[1] Torre LA, Siegel RL, Ward EM, Jemal A (2016). Global Cancer Incidence and Mortality Rates and Trends-An Update. Cancer Epidemiol Biomarkers Prev.

[2] Shelburne N, Simonds NI, Adhikari B, Alley M, Desvigne-Nickens P, Dimond E (2019). Changing Hearts and Minds: Improving Outcomes in Cancer Treatment-Related Cardiotoxicity. Curr Oncol Rep.

[3] Von Hoff DD, Layard MW, Basa P, Davis HL Jr, Von Hoff AL, Rozencweig M (1979). Risk factors for doxorubicin-induced congestive heart failure. Ann Intern Med.

[4] Lefrak EA, Pitha J, Rosenheim S, Gottlieb JA (1973). A clinicopathologic analysis of adriamycin cardiotoxicity. Cancer.

[5] Tomita S, Ishida M, Nakatani T, Fukuhara S, Hisashi Y, Ohtsu Y (2004). Bone marrow is a source of regenerated cardiomyocytes in doxorubicin-induced cardiomyopathy and granulocyte colony-stimulating factor enhances migration of bone marrow cells and attenuates cardiotoxicity of doxorubicin under electron microscopy. J Heart Lung Transplant.

[6] Ambler GR, Johnston BM, Maxwell L, Gavin JB, Gluckman PD (1993). Improvement of doxorubicin induced cardiomyopathy in rats treated with insulin-like growth factor I. Cardiovasc Res.

[7] Kuzu M, Kandemir FM, Yildirim S, Kucukler S, Caglayan C, Turk E (2018). Morin attenuates doxorubicin-induced heart and brain damage by reducing oxidative stress, inflammation and apoptosis. Biomed Pharmacother.

[8] Todorova VK, Siegel ER, Kaufmann Y, Kumarapeli A, Owen A, Wei JY (2020). Dantrolene Attenuates Cardiotoxicity of Doxorubicin Without Reducing its Antitumor Efficacy in a Breast Cancer Model. Transl Oncol.

[9] Szanto M, Rutkai I, Hegedus C, Czikora A, Rozsahegyi M, Kiss B (2011). Poly(ADP-ribose) polymerase-2 depletion reduces doxorubicin-induced damage through SIRT1 induction. Cardiovasc Res.

[10] Li DL, Wang ZV, Ding G, Tan W, Luo X, Criollo A (2016). Doxorubicin Blocks Cardiomyocyte Autophagic Flux by Inhibiting Lysosome Acidification. Circulation.

[11] Fang X, Wang H, Han D, Xie E, Yang X, Wei J (2019). Ferroptosis as a target for protection against cardiomyopathy. Proc Natl Acad Sci U S A.

[12] Wang L, Liu Y, Du T, Yang H, Lei L, Guo M (2020). ATF3 promotes erastin-induced ferroptosis by suppressing system Xc(.). Cell Death Differ.

[13] Zheng X, Zhong T, Ma Y, Wan X, Qin A, Yao B (2020). Bnip3 mediates doxorubicin-induced cardiomyocyte pyroptosis via caspase-3/GSDME. Life Sci.

[14] Li D, Yang Y, Wang S, He X, Liu M, Bai B (2021). Role of acetylation in doxorubicin-induced cardiotoxicity. Redox Biol.

[15] Christidi E, Brunham LR (2021). Regulated cell death pathways in doxorubicin-induced cardiotoxicity. Cell Death Dis.

[16] Zhang QL, Yang JJ, Zhang HS (2019). Carvedilol (CAR) combined with carnosic acid (CAA) attenuates doxorubicin-induced cardiotoxicity by suppressing excessive oxidative stress, inflammation, apoptosis and autophagy. Biomed Pharmacother.

[17] Zhou L, Li R, Liu C, Sun T, Htet Aung LH, Chen C (2017). Foxo3a inhibits mitochondrial fission and protects against doxorubicin-induced cardiotoxicity by suppressing MIEF2. Free Radic Biol Med.

[18] Ichikawa Y, Ghanefar M, Bayeva M, Wu R, Khechaduri A, Naga Prasad SV (2014). Cardiotoxicity of doxorubicin is mediated through mitochondrial iron accumulation. J Clin Invest.

[19] Deng S, Kruger A, Kleschyov AL, Kalinowski L, Daiber A, Wojnowski L (2007). Gp91phox-containing NAD(P)H oxidase increases superoxide formation by doxorubicin and NADPH. Free Radic Biol Med.

[20] Mu H, Liu H, Zhang J, Huang J, Zhu C, Lu Y (2019). Ursolic acid prevents doxorubicin-induced cardiac toxicity in mice through eNOS activation and inhibition of eNOS uncoupling. J Cell Mol Med.

[21] Nithipongvanitch R, Ittarat W, Cole MP, Tangpong J, Clair DK, Oberley TD (2007). Mitochondrial and nuclear p53 localization in cardiomyocytes: redox modulation by doxorubicin (Adriamycin)?. Antioxid Redox Signal.

[22] Brandes RP, Weissmann N, Schroder K (2014). Nox family NADPH oxidases: Molecular mechanisms of activation. Free Radic Biol Med.

[23] Efentakis P, Varela A, Chavdoula E, Sigala F, Sanoudou D, Tenta R (2020). Levosimendan prevents doxorubicin-induced cardiotoxicity in time- and dose-dependent manner: implications for inotropy. Cardiovasc Res.

[24] Tayeh Z, Ofir R (2018). Asteriscus graveolens Extract in Combination with Cisplatin/Etoposide/Doxorubicin Suppresses Lymphoma Cell Growth through Induction of Caspase-3 Dependent Apoptosis. Int J Mol Sci.

[25] McLaughlin D, Zhao Y, O'Neill KM, Edgar KS, Dunne PD, Kearney AM (2017). Signalling mechanisms underlying doxorubicin and Nox2 NADPH oxidase-induced cardiomyopathy: involvement of mitofusin-2. Br J Pharmacol.

[26] Ma ZG, Kong CY, Wu HM, Song P, Zhang X, Yuan YP (2020). Toll-like receptor 5 deficiency diminishes doxorubicin-induced acute cardiotoxicity in mice. Theranostics.

[27] Zhang X, Hu C, Kong CY, Song P, Wu HM, Xu SC (2020). FNDC5 alleviates oxidative stress and cardiomyocyte apoptosis in doxorubicin-induced cardiotoxicity via activating AKT. Cell Death Differ.

[28] Zhao Y, McLaughlin D, Robinson E, Harvey AP, Hookham MB, Shah AM (2010). Nox2 NADPH oxidase promotes pathologic cardiac remodeling associated with Doxorubicin chemotherapy. Cancer Res.

[29] Zhang M, Prosser BL, Bamboye MA, Gondim ANS, Santos CX, Martin D (2015). Contractile Function During Angiotensin-II Activation: Increased Nox2 Activity Modulates Cardiac Calcium Handling via Phospholamban Phosphorylation. J Am Coll Cardiol.

[30] Coats BR, Schoenfelt KQ, Barbosa-Lorenzi VC, Peris E, Cui C, Hoffman A (2017). Metabolically Activated Adipose Tissue Macrophages Perform Detrimental and Beneficial Functions during Diet-Induced Obesity. Cell Rep.

[31] Graham KA, Kulawiec M, Owens KM, Li X, Desouki MM, Chandra D (2010). NADPH oxidase 4 is an oncoprotein localized to mitochondria. Cancer Biol Ther.

[32] Zeng C, Duan F, Hu J, Luo B, Huang B, Lou X (2020). NLRP3 inflammasome-mediated pyroptosis contributes to the pathogenesis of non-ischemic dilated cardiomyopathy. Redox Biol.

[33] Lin J, Fang L, Li H, Li Z, Lyu L, Wang H (2019). Astragaloside IV alleviates doxorubicin induced cardiomyopathy by inhibiting NADPH oxidase derived oxidative stress. Eur J Pharmacol.

[34] Hu C, Zhang X, Zhang N, Wei WY, Li LL, Ma ZG (2020). Osteocrin attenuates inflammation, oxidative stress, apoptosis, and cardiac dysfunction in doxorubicin-induced cardiotoxicity. Clin Transl Med.

[35] Russo M, Guida F, Paparo L, Trinchese G, Aitoro R, Avagliano C (2019). The novel butyrate derivative phenylalanine-butyramide protects from doxorubicin-induced cardiotoxicity. Eur J Heart Fail.

[36] Akolkar G, Bagchi AK, Ayyappan P, Jassal DS, Singal PK (2017). Doxorubicin-induced nitrosative stress is mitigated by vitamin C via the modulation of nitric oxide synthases. Am J Physiol Cell Physiol.

[37] Bartesaghi S, Radi R (2018). Fundamentals on the biochemistry of peroxynitrite and protein tyrosine nitration. Redox Biol.

[38] Milano G, Biemmi V, Lazzarini E, Balbi C, Ciullo A, Bolis S (2020). Intravenous administration of cardiac progenitor cell-derived exosomes protects against doxorubicin/trastuzumab-induced cardiac toxicity. Cardiovasc Res.

[39] Gill SS, Tuteja N (2010). Reactive oxygen species and antioxidant machinery in abiotic stress tolerance in crop plants. Plant Physiol Biochem.

[40] Mukhopadhyay P, Rajesh M, Batkai S, Kashiwaya Y, Hasko G, Liaudet L (2009). Role of superoxide, nitric oxide, and peroxynitrite in doxorubicin-induced cell death *in vivo* and *in vitro*. Am J Physiol Heart Circ Physiol.

[41] Cole MP, Chaiswing L, Oberley TD, Edelmann SE, Piascik MT, Lin SM (2006). The protective roles of nitric oxide and superoxide dismutase in adriamycin-induced cardiotoxicity. Cardiovasc Res.

[42] Sukhovershin RA, Yepuri G, Ghebremariam YT (2015). Endothelium-Derived Nitric Oxide as an Antiatherogenic Mechanism: Implications for Therapy. Methodist Debakey Cardiovasc J.

[43] Topal G, Brunet A, Millanvoye E, Boucher JL, Rendu F, Devynck MA (2004). Homocysteine induces oxidative stress by uncoupling of NO synthase activity through reduction of tetrahydrobiopterin. Free Radic Biol Med.

[44] Zhang JX, Qu XL, Chu P, Xie DJ, Zhu LL, Chao YL (2018). Low shear stress induces vascular eNOS uncoupling via autophagy-mediated eNOS phosphorylation. Biochim Biophys Acta Mol Cell Res.

[45] Neilan TG, Blake SL, Ichinose F, Raher MJ, Buys ES, Jassal DS (2007). Disruption of nitric oxide synthase 3 protects against the cardiac injury, dysfunction, and mortality induced by doxorubicin. Circulation.

[46] Tedesco L, Rossi F, Ragni M, Ruocco C, Brunetti D, Carruba MO (2020). A Special Amino-Acid Formula Tailored to Boosting Cell Respiration Prevents Mitochondrial Dysfunction and Oxidative Stress Caused by Doxorubicin in Mouse Cardiomyocytes. Nutrients.

[47] Zeglinski M, Premecz S, Lerner J, Wtorek P, Dasilva M, Hasanally D (2014). Congenital absence of nitric oxide synthase 3 potentiates cardiac dysfunction and reduces survival in doxorubicin- and trastuzumab-mediated cardiomyopathy. Can J Cardiol.

[48] Dudka J, Burdan F, Korga A, Iwan M, Madej-Czerwonka B, Cendrowska-Pinkosz M (2012). Intensification of doxorubicin-related oxidative stress in the heart by hypothyroidism is not related to the expression of cytochrome P450 NADPH-reductase and inducible nitric oxide synthase, as well as activity of xanthine oxidase. Oxid Med Cell Longev.

[49] Krishnamurthy B, Rani N, Bharti S, Golechha M, Bhatia J, Nag TC (2015). Febuxostat ameliorates doxorubicin-induced cardiotoxicity in rats. Chem Biol Interact.

[50] Ashtar M, Tenshin H, Teramachi J, Bat-Erdene A, Hiasa M, Oda A (2020). The Roles of ROS Generation in RANKL-Induced Osteoclastogenesis: Suppressive Effects of Febuxostat. Cancers (Basel).

[51] Moruno-Manchon JF, Uzor NE, Kesler SR, Wefel JS, Townley DM, Nagaraja AS (2018). Peroxisomes contribute to oxidative stress in neurons during doxorubicin-based chemotherapy. Mol Cell Neurosci.

[52] do Nascimento TC, Cazarin CBB, Marostica MR Jr, Mercadante AZ, Jacob-Lopes E, Zepka LQ (2020). Microalgae carotenoids intake: Influence on cholesterol levels, lipid peroxidation and antioxidant enzymes. Food Res Int.

[53] Doroshow JH, Locker GY, Myers CE (1980). Enzymatic defenses of the mouse heart against reactive oxygen metabolites: alterations produced by doxorubicin. J Clin Invest.

[54] Zerikiotis S, Angelidis C, Dhima I, Naka KK, Kasioumi P, Kalfakakou V (2019). The increased expression of the inducible Hsp70 (HSP70A1A) in serum of patients with heart failure and its protective effect against the cardiotoxic agent doxorubicin. Mol Cell Biochem.

[55] Xiong Y, Liu X, Lee CP, Chua BH, Ho YS (2006). Attenuation of doxorubicin-induced contractile and mitochondrial dysfunction in mouse heart by cellular glutathione peroxidase. Free Radic Biol Med.

[56] Yen HC, Oberley TD, Gairola CG, Szweda LI, St Clair DK (1999). Manganese superoxide dismutase protects mitochondrial complex I against adriamycin-induced cardiomyopathy in transgenic mice. Arch Biochem Biophys.

[57] Yen HC, Oberley TD, Vichitbandha S, Ho YS, St Clair DK (1996). The protective role of manganese superoxide dismutase against adriamycin-induced acute cardiac toxicity in transgenic mice. J Clin Invest.

[58] Kang YJ, Chen Y, Epstein PN (1996). Suppression of doxorubicin cardiotoxicity by overexpression of catalase in the heart of transgenic mice. J Biol Chem.

[59] Doroshow JH, Esworthy RS, Chu FF (2020). Control of doxorubicin-induced, reactive oxygen-related apoptosis by glutathione peroxidase 1 in cardiac fibroblasts. Biochem Biophys Rep.

[60] Zare MFR, Rakhshan K, Aboutaleb N, Nikbakht F, Naderi N, Bakhshesh M (2019). Apigenin attenuates doxorubicin induced cardiotoxicity via reducing oxidative stress and apoptosis in male rats. Life Sci.

[61] Leung WS, Kuo WW, Ju DT, Wang TD, Shao-Tsu Chen W, Ho TJ (2020). Protective effects of diallyl trisulfide (DATS) against doxorubicin-induced inflammation and oxidative stress in the brain of rats. Free Radic Biol Med.

[62] Ma Q (2013). Role of nrf2 in oxidative stress and toxicity. Annu Rev Pharmacol Toxicol.

[63] Singh A, Venkannagari S, Oh KH, Zhang YQ, Rohde JM, Liu L (2016). Small Molecule Inhibitor of NRF2 Selectively Intervenes Therapeutic Resistance in KEAP1-Deficient NSCLC Tumors. ACS Chem Biol.

[64] Zhao L, Qi Y, Xu L, Tao X, Han X, Yin L (2018). MicroRNA-140-5p aggravates doxorubicin-induced cardiotoxicity by promoting myocardial oxidative stress via targeting Nrf2 and Sirt2. Redox Biol.

[65] Hu X, Liu H, Wang Z, Hu Z, Li L (2019). miR-200a Attenuated Doxorubicin-Induced Cardiotoxicity through Upregulation of Nrf2 in Mice. Oxid Med Cell Longev.

[66] Zhao Y, Sun J, Zhang W, Peng M, Chen J, Zheng L (2020). Follistatin-Like 1 Protects against Doxorubicin-Induced Cardiomyopathy through Upregulation of Nrf2. Oxid Med Cell Longev.

[67] Smith J (2002). Human Sir2 and the 'silencing' of p53 activity. Trends Cell Biol.

[68] North BJ, Marshall BL, Borra MT, Denu JM, Verdin E (2003). The human Sir2 ortholog, SIRT2, is an NAD+-dependent tubulin deacetylase. Mol Cell.

[69] Michishita E, Park JY, Burneskis JM, Barrett JC, Horikawa I (2005). Evolutionarily conserved and nonconserved cellular localizations and functions of human SIRT proteins. Mol Biol Cell.

[70] Hu C, Zhang X, Song P, Yuan YP, Kong CY, Wu HM (2020). Meteorin-like protein attenuates doxorubicin-induced cardiotoxicity via activating cAMP/PKA/SIRT1 pathway. Redox Biol.

[71] Yuan YP, Ma ZG, Zhang X, Xu SC, Zeng XF, Yang Z (2018). CTRP3 protected against doxorubicin-induced cardiac dysfunction, inflammation and cell death via activation of Sirt1. J Mol Cell Cardiol.

[72] Zheng W, Lu YB, Liang ST, Zhang QJ, Xu J, She ZG (2013). SIRT1 mediates the protective function of Nkx2.5 during stress in cardiomyocytes. Basic Res Cardiol.

[73] Sin TK, Tam BT, Yung BY, Yip SP, Chan LW, Wong CS (2015). Resveratrol protects against doxorubicin-induced cardiotoxicity in aged hearts through the SIRT1-USP7 axis. J Physiol.

[74] Pillai VB, Bindu S, Sharp W, Fang YH, Kim G, Gupta M (2016). Sirt3 protects mitochondrial DNA damage and blocks the development of doxorubicin-induced cardiomyopathy in mice. Am J Physiol Heart Circ Physiol.

[75] Cheung KG, Cole LK, Xiang B, Chen K, Ma X, Myal Y (2015). Sirtuin-3 (SIRT3) Protein Attenuates Doxorubicin-induced Oxidative Stress and Improves Mitochondrial Respiration in H9c2 Cardiomyocytes. J Biol Chem.

[76] Marechal X, Montaigne D, Marciniak C, Marchetti P, Hassoun SM, Beauvillain JC (2011). Doxorubicin-induced cardiac dysfunction is attenuated by ciclosporin treatment in mice through improvements in mitochondrial bioenergetics. Clin Sci (Lond).

[77] Sag CM, Kohler AC, Anderson ME, Backs J, Maier LS (2011). CaMKII-dependent SR Ca leak contributes to doxorubicin-induced impaired Ca handling in isolated cardiac myocytes. J Mol Cell Cardiol.

[78] You F, Gao C (2019). Topoisomerase Inhibitors and Targeted Delivery in Cancer Therapy. Curr Top Med Chem.

[79] Atwal M, Swan RL, Rowe C, Lee KC, Lee DC, Armstrong L (2019). Intercalating TOP2 Poisons Attenuate Topoisomerase Action at Higher Concentrations. Mol Pharmacol.

[80] Zhang S, Liu X, Bawa-Khalfe T, Lu LS, Lyu YL, Liu LF (2012). Identification of the molecular basis of doxorubicin-induced cardiotoxicity. Nat Med.

[81] Aung LHH, Li R, Prabhakar BS, Li P (2017). Knockdown of Mtfp1 can minimize doxorubicin cardiotoxicity by inhibiting Dnm1l-mediated mitochondrial fission. J Cell Mol Med.

[82] Coelho AR, Martins TR, Couto R, Deus C, Pereira CV, Simoes RF (2017). Berberine-induced cardioprotection and Sirt3 modulation in doxorubicin-treated H9c2 cardiomyoblasts. Biochim Biophys Acta Mol Basis Dis.

[83] Lee KH, Cho H, Lee S, Woo JS, Cho BH, Kang JH (2017). Enhanced-autophagy by exenatide mitigates doxorubicin-induced cardiotoxicity. Int J Cardiol.

[84] Tang F, Zhou X, Wang L, Shan L, Li C, Zhou H (2018). A novel compound DT-010 protects against doxorubicin-induced cardiotoxicity in zebrafish and H9c2 cells by inhibiting reactive oxygen species-mediated apoptotic and autophagic pathways. Eur J Pharmacol.

[85] Kawaguchi T, Takemura G, Kanamori H, Takeyama T, Watanabe T, Morishita K (2012). Prior starvation mitigates acute doxorubicin cardiotoxicity through restoration of autophagy in affected cardiomyocytes. Cardiovasc Res.

[86] Zheng K, Jiang Y, Liao C, Hu X, Li Y, Zeng Y (2017). NOX2-Mediated TFEB Activation and Vacuolization Regulate Lysosome-Associated Cell Death Induced by Gypenoside L, a Saponin Isolated from Gynostemma pentaphyllum. J Agric Food Chem.

[87] Cui N, Wu F, Lu WJ, Bai R, Ke B, Liu T (2019). Doxorubicin-induced cardiotoxicity is maturation dependent due to the shift from topoisomerase IIalpha to IIbeta in human stem cell derived cardiomyocytes. J Cell Mol Med.

[88] Lin RW, Ho CJ, Chen HW, Pao YH, Chen LE, Yang MC (2018). P53 enhances apoptosis induced by doxorubicin only under conditions of severe DNA damage. Cell Cycle.

[89] Mantawy EM, Esmat A, El-Bakly WM, Salah ElDin RA, El-Demerdash E (2017). Mechanistic clues to the protective effect of chrysin against doxorubicin-induced cardiomyopathy: Plausible roles of p53, MAPK and AKT pathways. Sci Rep.

[90] Zhu W, Zhang W, Shou W, Field LJ (2014). P53 inhibition exacerbates late-stage anthracycline cardiotoxicity. Cardiovasc Res.

[91] Chen Z, Zhu W, Bender I, Gong W, Kwak IY, Yellamilli A (2017). Pathologic Stimulus Determines Lineage Commitment of Cardiac C-kit(+) Cells. Circulation.

[92] Malliaras K, Zhang Y, Seinfeld J, Galang G, Tseliou E, Cheng K (2013). Cardiomyocyte proliferation and progenitor cell recruitment underlie therapeutic regeneration after myocardial infarction in the adult mouse heart. EMBO Mol Med.

[93] Sahu BD, Kumar JM, Kuncha M, Borkar RM, Srinivas R, Sistla R (2016). Baicalein alleviates doxorubicin-induced cardiotoxicity via suppression of myocardial oxidative stress and apoptosis in mice. Life Sci.

[94] Pacher P, Liaudet L, Bai P, Virag L, Mabley JG, Hasko G (2002). Activation of poly(ADP-ribose) polymerase contributes to development of doxorubicin-induced heart failure. J Pharmacol Exp Ther.

[95] Ali M, Kamjoo M, Thomas HD, Kyle S, Pavlovska I, Babur M (2011). The clinically active PARP inhibitor AG014699 ameliorates cardiotoxicity but does not enhance the efficacy of doxorubicin, despite improving tumor perfusion and radiation response in mice. Mol Cancer Ther.

[96] Burton TR, Gibson SB (2009). The role of Bcl-2 family member BNIP3 in cell death and disease: NIPping at the heels of cell death. Cell Death Differ.

[97] Mai FY, He P, Ye JZ, Xu LH, Ouyang DY, Li CG (2019). Caspase-3-mediated GSDME activation contributes to cisplatin- and doxorubicin-induced secondary necrosis in mouse macrophages. Cell Prolif.

[98] Tavakoli Dargani Z, Singla DK (2019). Embryonic stem cell-derived exosomes inhibit doxorubicin-induced TLR4-NLRP3-mediated cell death-pyroptosis. Am J Physiol Heart Circ Physiol.

[99] Singla DK, Johnson TA, Tavakoli Dargani Z (2019). Exosome Treatment Enhances Anti-Inflammatory M2 Macrophages and Reduces Inflammation-Induced Pyroptosis in Doxorubicin-Induced Cardiomyopathy. Cells.

[100] Miotto G, Rossetto M, Di Paolo ML, Orian L, Venerando R, Roveri A (2020). Insight into the mechanism of ferroptosis inhibition by ferrostatin-1. Redox Biol.

[101] Liu Y, Zeng L, Yang Y, Chen C, Wang D, Wang H (2020). Acyl-CoA thioesterase 1 prevents cardiomyocytes from Doxorubicin-induced ferroptosis via shaping the lipid composition. Cell Death Dis.

[102] Yang WS, SriRamaratnam R, Welsch ME, Shimada K, Skouta R, Viswanathan VS (2014). Regulation of ferroptotic cancer cell death by GPX4. Cell.

[103] Lakhal-Littleton S, Wolna M, Carr CA, Miller JJ, Christian HC, Ball V (2015). Cardiac ferroportin regulates cellular iron homeostasis and is important for cardiac function. Proc Natl Acad Sci U S A.

[104] Tadokoro T, Ikeda M, Ide T, Deguchi H, Ikeda S, Okabe K (2020). Mitochondria-dependent ferroptosis plays a pivotal role in doxorubicin cardiotoxicity. JCI Insight.

[105] Han D, Wang Y, Wang Y, Dai X, Zhou T, Chen J (2020). The Tumor-Suppressive Human Circular RNA CircITCH Sponges miR-330-5p to Ameliorate Doxorubicin-Induced Cardiotoxicity Through Upregulating SIRT6, Survivin, and SERCA2a. Circ Res.

[106] Yuan Y, Fan S, Shu L, Huang W, Xie L, Bi C (2020). Exploration the Mechanism of Doxorubicin-Induced Heart Failure in Rats by Integration of Proteomics and Metabolomics Data. Front Pharmacol.

[107] Holmgren G, Sartipy P, Andersson CX, Lindahl A, Synnergren J (2018). Expression Profiling of Human Pluripotent Stem Cell-Derived Cardiomyocytes Exposed to Doxorubicin-Integration and Visualization of Multi-Omics Data. Toxicol Sci.

[108] Wang X, Cheng Z, Xu J, Feng M, Zhang H, Zhang L (2021). Circular RNA Arhgap12 modulates doxorubicin-induced cardiotoxicity by sponging miR-135a-5p. Life Sci.

[109] Gupta SK, Garg A, Bar C, Chatterjee S, Foinquinos A, Milting H (2018). Quaking Inhibits Doxorubicin-Mediated Cardiotoxicity Through Regulation of Cardiac Circular RNA Expression. Circ Res.

[110] Yarana C, Carroll D, Chen J, Chaiswing L, Zhao Y, Noel T (2018). Extracellular Vesicles Released by Cardiomyocytes in a Doxorubicin-Induced Cardiac Injury Mouse Model Contain Protein Biomarkers of Early Cardiac Injury. Clin Cancer Res.

[111] Beer LA, Kossenkov AV, Liu Q, Luning Prak E, Domchek S, Speicher DW (2016). Baseline Immunoglobulin E Levels as a Marker of Doxorubicin- and Trastuzumab-Associated Cardiac Dysfunction. Circ Res.

[112] Huang KM, Zavorka Thomas M, Magdy T, Eisenmann ED, Uddin ME, DiGiacomo DF (2021). Targeting OCT3 attenuates doxorubicin-induced cardiac injury. Proc Natl Acad Sci U S A.

[113] Zhang L, Wang X, Feng M, Zhang H, Xu J, Ding J (2020). Peptidomics Analysis Reveals Peptide PDCryab1 Inhibits Doxorubicin-Induced Cardiotoxicity. Oxid Med Cell Longev.

